# Use of AI to assess COVID-19 variant impacts on hospitalization, ICU, and death

**DOI:** 10.3389/frai.2022.927203

**Published:** 2022-11-30

**Authors:** Waleed Hilal, Michael G. Chislett, Brett Snider, Edward A. McBean, John Yawney, S. Andrew Gadsden

**Affiliations:** ^1^Department of Mechanical Engineering, McMaster University, Hamilton, ON, Canada; ^2^School of Engineering, University of Guelph, Guelph, ON, Canada; ^3^College of Engineering, Mathematics, and Physical Sciences, University of Exeter, Exeter, United Kingdom; ^4^Adastra Corporation, Toronto, ON, Canada

**Keywords:** COVID-19, Delta variant, healthcare, hospitalization, medical risk factors, Omicron variant, XGBoost

## Abstract

The rapid spread of COVID-19 and its variants have devastated communities worldwide, and as the highly transmissible Omicron variant becomes the dominant strain of the virus in late 2021, the need to characterize and understand the difference between the new variant and its predecessors has been an increasing priority for public health authorities. Artificial Intelligence has played a significant role in the analysis of various facets of COVID-19 since the early stages of the pandemic. This study proposes the use of AI, specifically an XGBoost model, to quantify the impact of various medical risk factors (or “population features”) on the possibility of a patient outcome resulting in hospitalization, ICU admission, or death. The results are compared between the Delta and Omicron COVID-19 variants. Results indicated that older age and an unvaccinated patient status most consistently correspond as the most significant population features contributing to all three scenarios (hospitalization, ICU, death). The top 15 features for each variant-outcome scenario were determined, which most frequently included diabetes, cardiovascular disease, chronic kidney disease, and complications of pneumonia as highly significant population features contributing to serious illness outcomes. The Delta/Hospitalization model returned the highest performance metric scores for the area under the receiver operating characteristic (AUROC), F1, and Recall, while Omicron/ICU and Omicron/Hospitalization had the highest accuracy and precision values, respectively. The recall was found to be above 0.60 in most cases (with only two exceptions), indicating that the total number of false positives was generally minimized (accounting for more of the people who would theoretically require medical care).

## 1. Introduction

The characterization of SARS-CoV-2 risk factors has been at the forefront of pandemic research since the onset of COVID-19 (Rod et al., 2020). While trends in public health data provide valuable insight into the understanding of the attributes associated with severe COVID-19 illness, artificial intelligence (AI) provides a dynamic, computational, and quantifiable approach to describing the features of a population putting them at higher risk of serious sickness. Specifically, those experiencing harsher symptoms are more likely to become hospitalized, admitted to an intensive care unit (ICU), or face possible mortality. As global deaths from COVID-19 surpass 5.5 million in late January 2022, in the face of a newer and more transmissible variant of concern (VOC), anticipating deaths is essential to prepare health care systems and jurisdictions around the world (World Health Organization, 2022).

The merits of advising the decision-making processes during the pandemic have been amplified by the use of AI to better describe the significance of various medical risk factors (Vaishya et al., 2020). Algorithms designed to assess the complicated nature of COVID-19 illness can articulate the complexity of underlying medical conditions and demographic information, and inform public health agencies on how these features may significantly influence the trajectory of a patient's experience with the virus. While epidemiological and infectivity modeling (e.g., SEIR and SIR modeling) have been employed strategically to characterize population dynamics throughout the pandemic (Yawney and Gadsden, 2020), AI presents a unique methodology to critically interpret medical risk factors.

An eXtreme Gradient Boosting (XGBoost) modeling approach is proposed herein as a reliable, consistent, and accurate method for achieving the quantified impact assessment of various population features and conditions. Specifically, with the less charted territory of COVID-19 variant research, the modeling described herein uses an Ontario-wide collection of positivity data to generate XGBoost models for three scenarios of outcome (hospitalization, ICU admittance, and death) for two unique variants (Delta and Omicron). This constitutes six distinct sets of modeling results, illustrating the attributes of a population that are more likely to lead a patient to one of the defined scenario outcomes.

The objective of this study is to describe and evaluate various population features using AI, ordering them by importance, to better understand the difference in the risk factors that are associated with severe illness in the Delta variant vs. the Omicron variant of COVID-19. The novelty of applying AI (specifically XGBoost) during the time of rising Omicron cases establishes this investigation as an entry into the forefront of pandemic research. This article describes published research in the cross-sections of AI and COVID-19 (Section 2), a detailed methods and procedures protocol for manipulation of the Ontario dataset (Section 3), results from each of the variant-scenario models (Section 4), and a discussion providing insights into the significance of the resultant top features (Section 5). A discussion of the limitations and performance metrics of the developed models is also provided. The impact assessment generated by the results of these models provides key messaging on the factors influencing illness associated with the new Omicron variant, allowing for the decisive allocation of hospitalized care resources.

## 2. Purposing AI to characterize COVID-19: A review

From the outset of the COVID-19 pandemic, novel publications in the areas of epidemiology, infectious diseases, and public health have been at the forefront of the research realm. Among these fields of interest have been advancements and unique applications of artificial intelligence, machine learning, computational modeling, and prediction methods to characterize, describe, and suggest implications of the various aspects of the pandemic.

Medically, AI has been applied throughout COVID-19 to establish diagnosis methods, using chest CAT Scan data and a neural network AI model to distinguish characteristics of COVID-19 from patients with influenza type A, influenza type B, pneumonia, and non-pneumonia (Jin et al., 2020). The approach to implementing AI and machine learning in COVID-19 diagnosis through computer tomography was widely published and has been reported as a contender to radiology-based diagnosis (Chen et al., 2020; Li L. et al., 2020; Wang et al., 2020).

Forecasts and predictions of cumulative cases have also been conducted using AI. Neural Network frameworks have anticipated next-day COVID-19 cases, on the basis of cumulative cases in the 5 days prior, the total cases historically, the total recovered cases, and the cumulative deaths (Huang et al., 2020). Similar forecasting anticipated the top 15 countries for new cases in 2020, using a machine learning autoregressive integrated moving average model, which correctly predicted that the United States would become the epicenter for the virus by mid-2020 (Kumar et al., 2020). A very similar application with matching results used the Eureqa AI model (Li M. et al., 2020).

The necessity to understand factors dictating the triage of patients was addressed in a study outlining those who should receive priority hospitalization based on a series of AI models (Neural Networks, Random Forest, and more) assessing (as features) the impact of various symptoms, underlying medical conditions, and demographics (Pourhomayoun and Shakibi, 2021). Age, gender, respiratory distress, diabetes, hypertension, and kidney disease were listed as the features corresponding most frequently with patient death (Pourhomayoun and Shakibi, 2021).

### 2.1. Related studies in Ontario, Canada

An application of different AI models (XGBoost, Neural Network, Random Forest) to predict the possibility of death from COVID-19 compared various population characteristics as features, and the degree to which each feature influences a patient's future mortality (Snider et al., 2020). This research found that XGBoost, the same model used in this study, is a reliable method to train COVID-19 cases for death-based predictions, with the highest degree of precision and accuracy (Snider et al., 2020).

A second study following (Snider et al., 2020) had similar results; from the same three AI models, age is presented as the most important feature contributing to COVID-19 mortality (Snider et al., 2021a). Also under consideration in the XGBoost model were several features overlapping with those described in this paper, namely dementia, hypertension, diabetes, kidney disease, and cancer, among others (Snider et al., 2021a).

Another application of XGBoost interpreted various risk factors and concluded similar findings to the initial AI studies, specifically that age is the most significant contributor to mortality (Snider et al., 2021b). An added dimension to this study focused on the implication of socioeconomic conditions, and how patients from low-income and ethnically concentrated regions are more likely to be associated with COVID-19 fatality (Snider et al., 2021b).

### 2.2. Variants of concern and AI

The emergence in late 2021 of the Omicron variant was accompanied by new reports that three doses of a COVID-19 vaccine will result in a lower likelihood of becoming symptomatic (regardless of the mutated strain's greater rate of infection) (Accorsi et al., 2022). A key barrier in the fight against the new variant of concern is the insufficient uptake of vaccinations in the western world, causing a strain on hospitals and health care workers (del Rio et al., 2022). As management of the health care system becomes increasingly concerning in an “endemic” phase of COVID-19, those who are unvaccinated pose a serious risk to themselves and others as the most likely patients (next to the elderly) to become seriously ill (Johnson et al., 2022).

Omicron is reportedly more capable of penetrating immune systems, with transmissibility remarkably higher (2–3 times more) than the previous dominant strain, Delta (del Rio et al., 2022). It has also been reported that Omicron could be 10 times more transmissible than the original SARS-CoV-2 strain (Al-Tawfiq et al., 2022). While it has been studied that two doses of a vaccine can help protect a population, a booster or third dose can prove beneficial during the peak of the Omicron wave of new cases (Andrews et al., 2022).

The projected outlook on the pandemic has more recently evolved from a concern of infection prevention to severity prevention, and studies out of South Africa show that during the Omicron wave experienced by that country, at an earlier point than Canada and the United States, there has been a “decoupling” of hospitalization and deaths (Madhi et al., 2022). The decoupling of the incidence rates suggests that while Omicron is likely to ravage communities much more frighteningly than Delta, this is not likely to be accompanied by unfathomable death rates (Madhi et al., 2022). Hence, as the mutations evolve into less severe yet more transmissible infections, health care systems will experience an increased demand for patient care and a new challenge from the initial months of the pandemic.

In artificial intelligence practices, studies on the variants of concern and their associated symptoms have been less explored than other COVID-19 research in computational sciences. A single focus has arisen as the dominant concern for modeling applications throughout the onset of the Omicron variant; namely, the introduction of AI as an advisory tool to make health care-related decisions to reduce the stress on an overworked, under-resourced system (Nadeem et al., 2022). X-Ray and computer tomography images, as seen in the earlier stages of the pandemic, are once again being combined with deep learning and artificial intelligence models to detect specific variants, including Omicron (Khan et al., 2022).

The limited scope, as identified to date for AI-based assessments of the COVID-19 variants, is now emerging as a research area essential for the characterization and understanding of the virus' complexity, and the new endemic phase of the novel coronavirus.

## 3. Methodology

Data used in this AI analysis of variant impacts on hospitalization, ICU, and death was feasible using an Ontario population data package, consisting of individuals who were tested for COVID-19 from a period of March 2020 until late December 2021. These data were sourced from ICES under an agreement to conduct COVID-19 research in Canada. Datasets were linked using unique encoded identifiers and analyzed at ICES. Privacy legislation in Ontario permits, without individual consent, the study and general use of the province's health care data, which is intended for the assessment of the health care system and the safeguarding of public health.

Patient data consisted of an initial population size of 1,018,189 persons. After data cleaning (detailed in this section), the population was reduced to a size of 608,140 persons.

In this study, an XGBoost modeling method is integrated with the data to characterize three potential scenarios of patient outcomes: hospitalization, requirement of intensive care, or death. From this analysis, SHapley Additive exPlanations (SHAP) values are generated to indicate the importance and impact of the various features contributing to rates of all three scenarios.

Prior to implementing the XGBoost model, a modification of the dataset was eliminated and prepared the data for use. First, entries associated with an unknown health outcome (not definitively corresponding to hospitalized, admitted to ICU, or fatality) were omitted from the analysis. Also dropped were any entries with an absent value for age.

Since active cases of the virus could potentially cause a misrepresentation of the analysis results, entries associated with individuals still in the hospital or not yet recovered from COVID-19-related illness were excluded from the dataset. Following the data cleaning, various conditions or “features” were included. These features represent the characteristics of any given patient and are used to predict the dominating causes for increased rates of hospitalization, ICU admittance, and death. All initial features are listed in Table 1.

**Table 1:** Master list of patient features, prior to filtering, and hyperparameter tuning.

Acute respiratory distress syndrome	Age at time of illness	Anemia	Arrhythmia
Asthma	Asymptomatic	Cancer History	Cancer Ongoing
Cardiac Disease	Cardiovascular Disease	Chronic Diabetes	Chronic Asthma
Chronic Congestive Heart Failure	Chronic Dementia	Chronic Human Immunodeficiency Virus	Chronic Hypertension
Chronic Kidney Disease	Chronic Obstructive Pulmonary Disease	Chronic Obstructive Pulmonary Disease History	Chronic Rheumatoid Arthritis
Cirrhosis	Complex Continuing Care	Direct Exposure Cirrhosis	Encephalitis
Heart Failure	Immunocompromised	Liver Disease	Liver Failure
Local Health Integration Network	Multisystem Inflammatory Syndrome	Neurological Disorder	Obesity
Other Complications	Other Medical Risk Factors	Pneumonia	Postpartum
Pregnant	Previously COVID Positive	Public Health Unit	Renal Disease
Renal Failure	Respiratory Failure	Sepsis	Sex
Socioeconomic Homeless	Socioeconomic Rural	Symptom Cough	Symptom Fatigue
Symptom Fever	Symptom Headache	Symptom Other	Symptom Shortness of Breath
Symptom Sore Throat	Travel Within 14 Days	Tuberculosis	Underlying Medical Condition
Vaccination	Variant of Concern		

Due to the unique population representation in the provided dataset, all data associated with individuals who had a chronic or “diagnosed” condition are combined with those who had “self-reported” conditions. As an example, this was applied to the population identified as having diabetes (among others).

Distinct datasets were created from the master data to split the population by COVID-19 variant type. For this modification, patients reported as having contracted the Delta VOC were grouped, and all patients associated with the Omicron VOC were separated from the Delta group. All vaccination statuses (one or multiple doses of Pfizer, Moderna, and/or AstraZeneca) were grouped into the single variable of “vaccinated,” which was compared with all patients who were not vaccinated.

Following the distinction of the two new datasets, specific features were assessed and dropped based on a variance inflation factor (VIF) analysis. Features corresponding to relatively high collinearity were eliminated from the features of consideration. For this analysis, a VIF greater than 1.5 is considered to be the threshold for high collinearity, and any feature exceeding this VIF was eliminated.

Since the purpose of the analysis is to characterize contributing factors in COVID-19 hospitalization, ICU entry, and death, these descriptors of the data provided are converted from features into labels, such that the XGBoost model would not take these components into account.

Where *X* is the analysis data, and *Y* is the associated labels, a 70/30 training-testing split on the *X* and *Y* values is executed. In place of a conventional grid search approach to tune for hyperparameters, a more efficient randomized search method was used to tune the model. This was undertaken for parameters such as maximum depth, learning rate, and scale positive weight, among others. A StratifiedKFold cross-validation method is also implemented within the model to generate additional training sets. Three folds (train on two, test on one) were aimed at reducing the overfitting of the model and dataset, minimizing the training error, and increasing testing error. This was undertaken for all datasets.

Logistic loss (a loss function) was used in this model to fulfill the evaluation and consideration of prediction-based accuracy. This loss function has been most simply described by Equation 1 (Wu et al., 2021).


(1) $$L(y_{k},{\hat{y}}_{k})=((1-y_{k})\ln(1+e^{-{\hat{y}}_{k}}))+y_{k}\ln(1+e^{-{\hat{y}}_{k}})$$

Here, *y*_*k*_ is the *k*^*th*^ “real” value, and ${\hat{y}}_{k}$ is the *k*^*th*^ “predicted” value from the model.

The best hyperparameters are put forward to the XGBoost classifier, where the model trains using the provided hyperparameters to make final predictions. The resultant predictions are detailed in the following section. The filtered list of features, following the removal of various features from the collinearity analyses, is presented in Table 2.

**Table 2:** The filtered list of patient features, with modified vaccination statuses and high collinearities removed.

Acute respiratory distress syndrome	Age at time of illness	Anemia	Arrhythmia	Asthma
Asymptomatic	Cancer History	Cancer Ongoing	Cardiac Disease	Cardiovascular Disease
Chronic Diabetes	Chronic Congestive Heart Failure	Chronic Dementia	Chronic Human Immunodeficiency Virus	Chronic Kidney Disease
Chronic Obstructive Pulmonary Disease	Chronic Obstructive Pulmonary Disease History	Chronic Rheumatoid Arthritis	Cirrhosis	Complex Continuing Care
Direct Exposure Cirrhosis	Encephalitis	Heart Failure	Immunocompromised	Liver Disease
Liver Failure	Multisystem Inflammatory Syndrome	Neurological Disorder	Obesity	Other Complications
Other Medical Risk Factors	Pneumonia	Postpartum	Pregnant	Previously COVID Positive
Renal Disease	Renal Failure	Respiratory Failure	Sepsis	Socioeconomic Homeless
Symptom Fatigue	Symptom Fever	Symptom Other	Tuberculosis	Underlying Medical Condition
Unvaccinated/Vaccinated				

## 4. Results

XGBoost results have been generated in four formats; a SHapley Additive exPlanations (SHAP) value plot to illustrate the relative impacts of each feature on the likelihood of each scenario (hospitalization, ICU, death), performance metrics to describe the suitability of the model and comparative accuracy, bar graphs to show the absolute impacts of the top 15 features, and scatter plots representing the significance of select key model features.

### 4.1. Hospitalization

**Figure 1:**
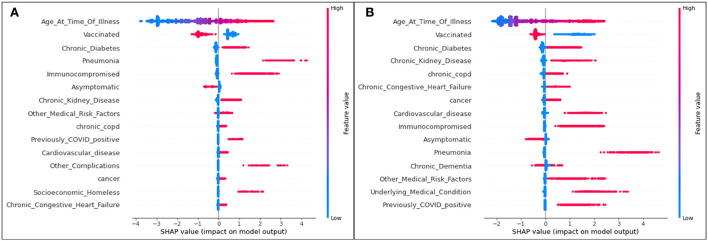
SHAP plots for **(A)** Delta and **(B)** Omicron features contributing to rates of hospitalization.

The plot summarizing SHAP values for the hospitalization analysis is provided in Figure 1.

For both variants, age is the most significant feature influencing one's possibility of being admitted to the hospital due to COVID-19 illness. As the only continuous variable, the SHAP entry for age illustrates that the older the patient (the redder the marker), the more likely they are to enter the hospital (the more positive the SHAP value). All other features are discrete variables, and thus red marks represent an affirmative case (the patient *does* identify with the defined feature) and blue marks represent a dissident case (the patient *does not* identify with the defined feature). Positive SHAP values dictate that the defined case is a contributor to hospitalization (hence, a blue marker with a positive SHAP value means that a patient not identifying with the feature is more likely to be admitted to the hospital than a patient who does identify with the feature).

The second most significant feature in both variants for hospitalization is an unvaccinated patient status. In both cases, it is predicted that those who are unvaccinated, regardless of the variant, are more likely to be hospitalized than those who are vaccinated.

Although their positions differ slightly (with the higher features representing more significant characteristics of a population), the list of the top 15 features mostly overlaps for Delta and Omicron cases.

**Figure 2:**
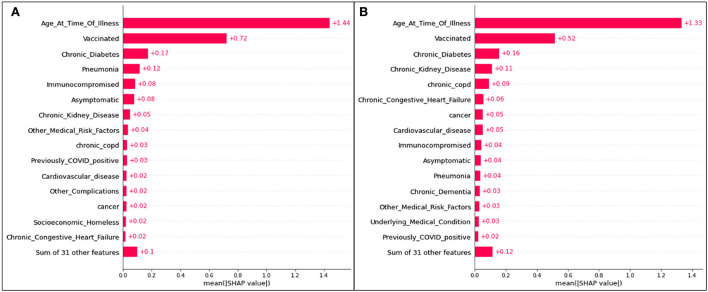
Absolute SHAP impact values for **(A)** Delta and **(B)** Omicron features contributing to hospitalization.

Bar plots presenting the absolute impacts of the top 15 features, and the difference from one feature to another, in hospitalization predictions, is displayed in Figure 2.

Figure 2 does not suggest whether or not identifying with a particular feature will lead to hospitalization; however, the average absolute SHAP values indicate the level of significance of a given feature and illustrates the difference between each of the top 15 features. The collective weight of the remaining features outside of the top 15 is also presented.

For Delta case hospitalization, age has a mean SHAP value of 1.41, a significant difference from the rest of the top features (even from the second highest value, associated with unvaccinated patients at 0.56). After the fourth top feature (complications of pneumonia), features are less distinguishable from one another and have very similar (and for several features, equal) absolute SHAP values. Hence, it can be inferred that, for this analysis, most features (five through fifteen) are no more impactful than others, but age, vaccination status, diabetes, and pneumonia play the greatest roles.

For Omicron case hospitalization, similar trends are noticed, with an age mean SHAP value of 1.33, and other features predicted to be much less impactful. An unvaccinated status had a relative impact of 0.51 (similar to the Delta cases), and after the fourth and fifth features (diabetes and chronic kidney disease), most features are comparable in impact.

**Figure 3:**
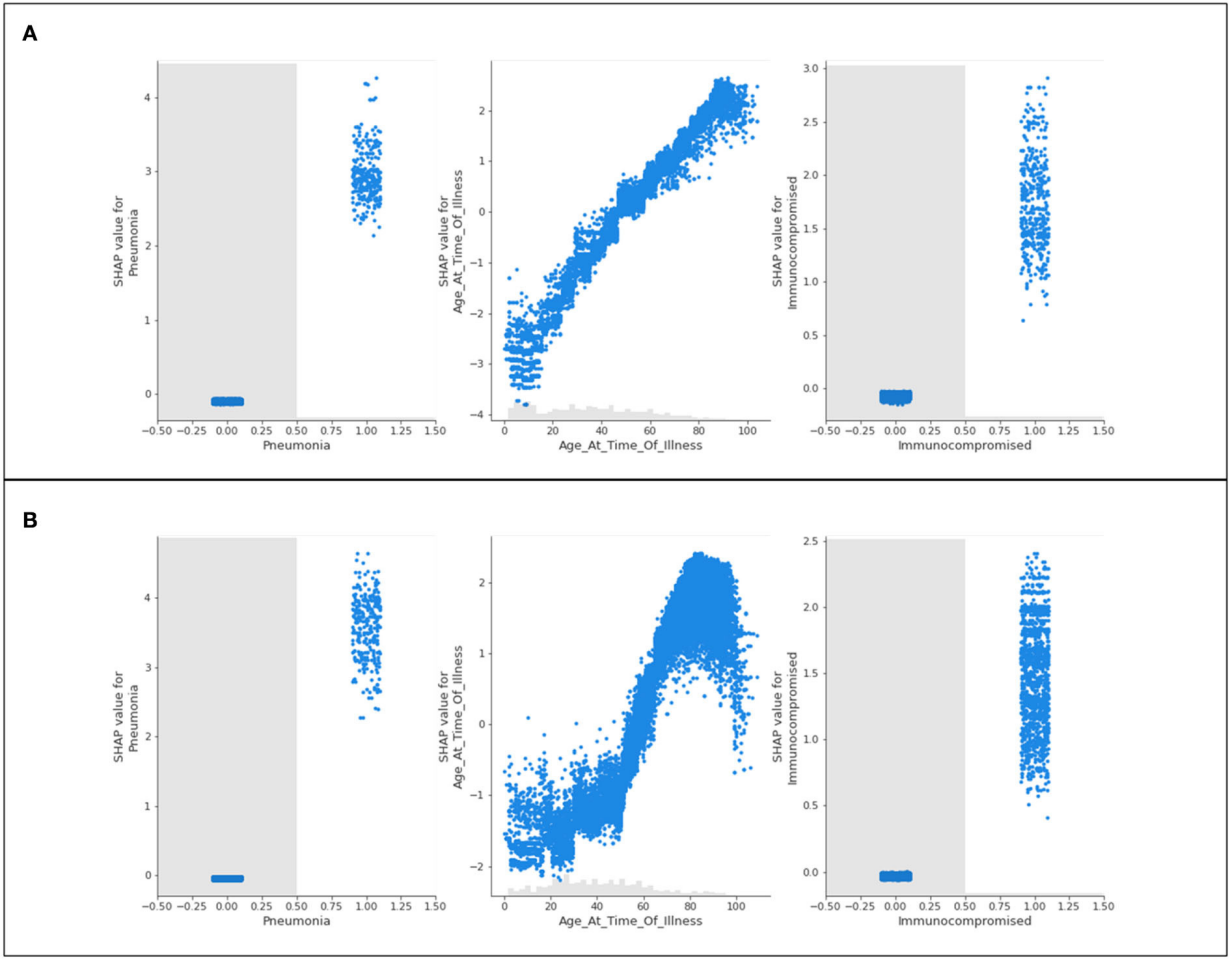
Scatter plots of three features of concern (pneumonia, age, and immunocompromised) for **(A)** Delta- and **(B)** Omicron- related hospitalization.

Three features of concern are selected for further analysis into their impacts on a population's likelihood to be hospitalized; complications of pneumonia, age, and a medical risk factor classified as immunocompromised. Scatter plots presenting the distribution of predicted data for the three features of concern are presented in Figure 3.

Figure 3 shows nearly identical trends for Delta and Omicron hospitalization predictions. For those who are immunocompromised or have complications associated with pneumonia, most patients have a high likelihood of being hospitalized. Conversely, those who do *not* have either of these classifications are neither more nor less likely to be hospitalized (not being immunocompromised, or not having complications of pneumonia will not influence hospitalization predictions). The general trend for age shows that younger populations are not predicted to be hospitalized, whereas older populations are predicted to be hospitalized. This is with the exception of “extreme” age classifications (approximately 100 years and older) where there is a steep drop in the prediction of hospitalization (a discussion of this phenomenon is provided in Section 5).

### 4.2. Intensive care unit

**Figure 4:**
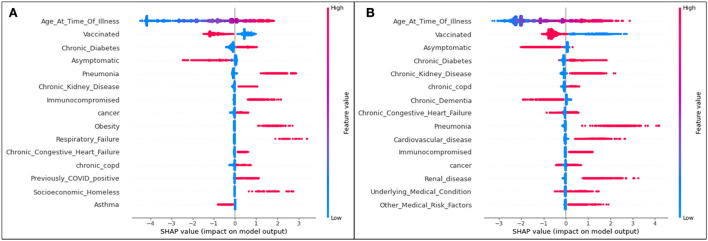
SHAP plots for **(A)** Delta and **(B)** Omicron features contributing to rates of ICU admittance.

The plot summarizing SHAP values for the ICU analysis is provided in Figure 4.

Aligning with the results for the hospitalization results, for both variants, age is the most significant feature influencing one's possibility of being admitted to the ICU due to COVID-19 illness. The second most significant feature in both variants for both hospitalization and ICU as seen in Figure 4 is an unvaccinated patient status. In all cases, unvaccinated patients are predicted to be more likely to become admitted to the ICU (and therefore have a more serious illness). Again, it is seen that, while specific ordering differs slightly from one feature to another, the top 15 features are mostly matched for Delta and Omicron ICU predictions.

**Figure 5:**
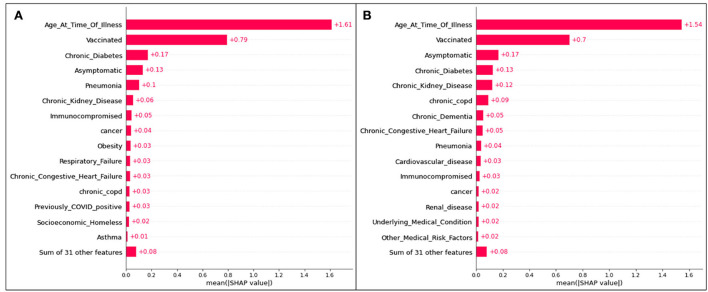
Absolute SHAP impact values for **(A)** Delta and **(B)** Omicron features contributing to ICU predictions.

Bar plots presenting the absolute impacts of the top 15 features, and the difference from one feature to another, in ICU predictions, are displayed in Figure 5.

For Delta cases in ICU, age has a mean SHAP value of 1.49, which (similar to hospitalization predictions) is much greater than the second leading feature, an unvaccinated status at an average absolute SHAP value of 0.54. Almost identically to Delta cases predicted to be hospitalized in Figure 2, after the fourth and fifth top features (complications of pneumonia and asymptomatic carriers, respectively), features are relatively indistinguishable, and are similar or equal in SHAP values. Therefore, age, vaccination status, diabetes, and pneumonia are once again the most prominent characteristics of predicted ICU admittance (Delta cases).

For Omicron ICU cases, the results are very similar to those seen in the Omicron hospitalization bar plot, with (in decreasing order) age, unvaccinated status, asymptomatic carriers, diabetes, and chronic kidney disease ranking as the most impactful features in model predictions. Age has the highest mean SHAP value of 1.54, with the unvaccinated averaging at 0.70.

**Figure 6:**
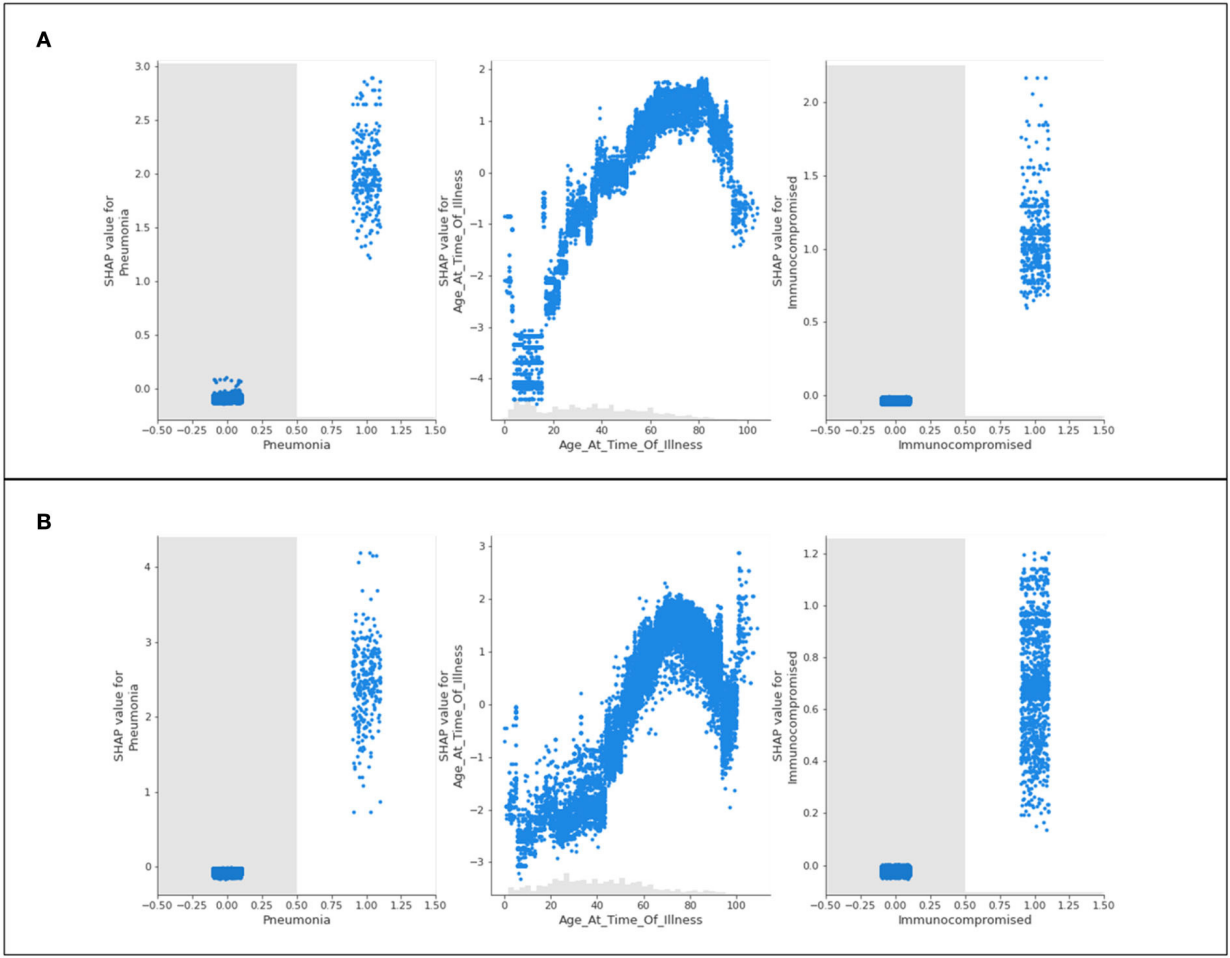
Scatter plots of three features of concern (pneumonia, age, and immunocompromised) for **(A)** Delta- and **(B)** Omicron- related ICU admittance predictions.

The features of concern (complications of pneumonia, age, and a medical risk factor classified as immunocompromised) are once again presented as scatter plots in Figure 6.

The recurring trend that was noted for hospitalization predictions is displayed in Figure 6. The features of concern for Delta and Omicron are akin in behavior, where those who are immunocompromised or have complications of pneumonia are predicted to most likely be admitted to ICU, whereas patients who do not identify with either of these characteristics are not influenced by them whatsoever (not being immunocompromised does not prevent ICU predictions, as an example). Again, the age scatter plot shows that younger populations are not predicted to enter the ICU, and aging patients are predicted to develop a sufficiently serious illness to substantiate intensive care. The exception of the extremely elderly population is once again noted and discussed in Section 5.

### 4.3. Death

**Figure 7:**
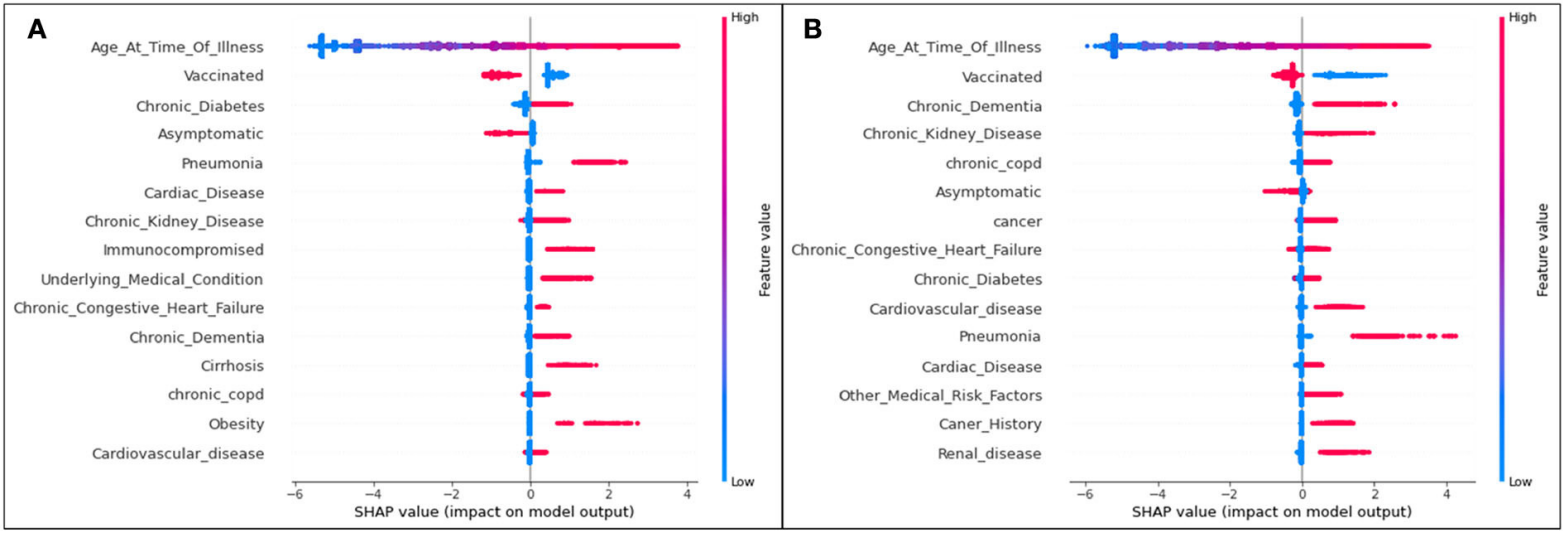
SHAP plots for **(A)** Delta and **(B)** Omicron features contributing to prediction of death from COVID-19.

The plot summarizing SHAP values for the death analysis is provided in Figure 7.

The trends of the death prediction SHAP plot are aligned with the results of both hospitalization and ICU. Age is once again the most significant feature influencing one's possibility of dying from COVID-19, followed by being unvaccinated against the virus. This tendency is (as seen in the other results) the same in both Delta and Omicron predictions. While the top 15 Delta and Omicron features had very similar overlaps in the hospitalization and ICU analyses (with the exception of few different features), the death analysis shows several different features for the two variants.

**Figure 8:**
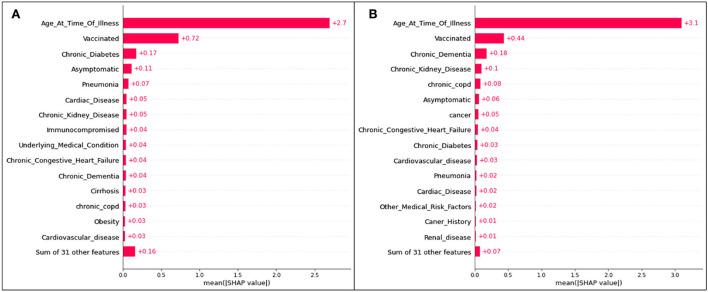
Absolute SHAP impact values for **(A)** Delta and **(B)** Omicron features contributing to death predictions.

The death-related top 15 features' absolute impacts are presented in the bar plot Figure 8.

For predictions of death, age has the largest mean SHAP value of all scenarios, reaching 2.7 for Delta cases and 3.1 for Omicron cases. These values are extremely high relative to the other features, with vaccination status (once again the second highest SHAP value) reaching values of 0.72 (Figure 8A) and 0.44 (Figure 8B).

**Figure 9:**
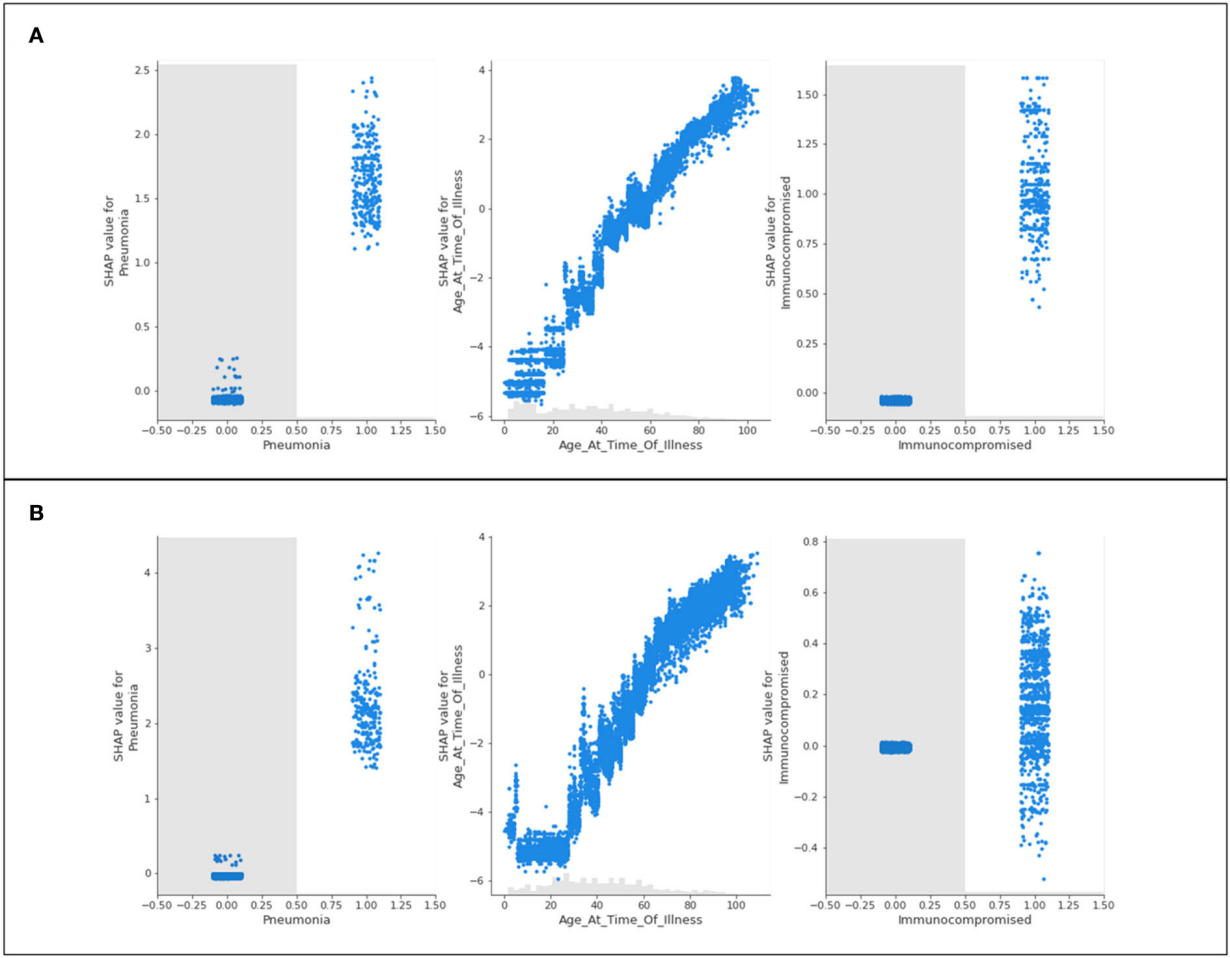
Scatter plots of three features of concern (pneumonia, age, and immunocompromised) for **(A)** Delta- and **(B)** Omicron- related death predictions.

The features of concern (complications of pneumonia, age, and a medical risk factor classified as immunocompromised) are once again presented as scatter plots in Figure 9.

The features of concern for Delta and Omicron are once again similar in their behavior to the presentation of results for hospitalization and intensive care. Immunocompromised patients and patients experiencing complications of pneumonia are more likely to be predicted as terminal patients. Those who do not identify with either feature are not influenced by their predictions. The age scatter plot predicts that younger populations will not likely die from the variants, and aging patients are more likely to result in fatality. Unlike the trend seen in hospitalization and ICU predictions, a steep and sudden drop in the SHAP value scatter plots for age is not noted in the extremely high ages.

### 4.4. Performance metrics

The performance metrics for all illness analyses (both variants) are provided in Table 3.

**Table 3:** Performance metrics for XGBoost analysis of Delta and Omicron COVID-19 analyses of hospitalization, ICU admittance, and death.

		**AUROC**	**F1 Score**	**Accuracy**	**Recall**	**Precision**
Delta	Hospitalization	**0.81**	**0.35**	0.85	**0.77**	0.23
	ICU	0.77	0.25	0.95	0.60	0.15
	Death	0.78	0.27	0.96	0.60	0.17
Omicron	Hospitalization	0.78	0.34	0.94	0.61	**0.23**
	ICU	0.57	0.15	**0.99**	0.13	0.18
	Death	0.70	0.27	0.98	0.42	0.20

Maximum values of each metric are emphasized in boldface.

The bolded values in the table indicate the model that provided results with the highest achieved performance metric in that defined category. Hence, the hospitalization predictions for Delta cases have the highest achieved AUROC, F1 score, and recall, whereas ICU predictions and hospitalization predictions in Omicron cases have the highest accuracy and precision scores, respectively.

## 5. Discussion

In all studied scenarios (hospitalization, ICU, and death), the top 15 features contributing to each prediction are noted with significant overlap. In most cases, the features in Delta variant predictions align with those of the Omicron variant predictions. However, the ordering of these features (and therefore, their level of impactfulness) differs between variants.

Consistently, Delta predictions had the top features (after age and unvaccinated status) of diabetes, asymptomatic carriers, and pneumonia. For the Omicron predictions, the top features were consistently chronic kidney disease and chronic obstructive pulmonary disease. This was also sporadically accompanied by features regarding dementia and diabetes. A difference in the ordering was also noted in the lower-tier features (those whose significance ranks in the top 15, but not very high in ranking). For Delta predictions, these features were cancer and a socioeconomic status “homeless” for hospitalization and ICU, and cardiovascular disease and obesity for death.

These key differences speak to the campaigning of many public health organizations in early 2022: reports of Omicron cases being highly transmissible, but not causing severe illness when compared to the Delta variant (Vaughan, 2021; Vilches et al., 2021). Hence, while common chronic illnesses debilitate the Delta population more than the Omicron population (who are more impacted if they identify with pulmonary and less common chronic diseases), it can be inferred that Delta may cause severe impacts on a broader population, and those with less common but more severe medical conditions should anticipate more severe illness than the average patient if contracting the Omicron variant.

Despite the significant differences in the variants (from risk factors to transmissibility), model predictions consistently report that unvaccinated patients (in comparison with those who had received vaccinations) are highly susceptible to serious health repercussions from COVID-19. The model suggests that an unvaccinated patient status is the second leading reason for hospitalization, admission to intensive care, and death for patients of either COVID-19 Delta or Omicron variants. On average, the absolute SHAP value (suggested overall impact) difference between the unvaccinated feature and the third most impactful feature for all models is 0.42. This large gap is exacerbated when considering that the majority of the top 15 features in all models have mean SHAP values between 0.02 and 0.05, generally. This wide difference articulates the modeled significance of patients identifying as unvaccinated against COVID-19.

The results consistently indicate that age plays a significant factor in a patient's likelihood of experiencing the worst possible outcomes from COVID-19. In all cases, the younger the patient results in the lesser likelihood of becoming hospitalized, receiving admittance to intensive care, or becoming terminally ill. Oppositely, older patients are predicted to at a higher likelihood of deteriorating more substantially (regardless of the variant) and requiring hospital care. The oldest patients are also predicted to be more likely to die as a result of the virus, whereas the younger populations (under the age of 40) are almost never predicted to have fatalities.

In all age scatter plots for hospitalization and ICU (Figures 3, 6), while increasing age has a steadily increasing SHAP value (indicating a higher prediction of needing care), there are steep “drops” in the plots after 90 years of age (and commonly at or around 100 years of age) where the high value in age is associated with a large reduction in the SHAP value. This implies that 90–100-year-old patients are not likely to be hospitalized or admitted to ICU. This result should not be misinterpreted as a less severe illness; this trend is not identified for the scatter plots associated with death predictions (Figure 9). In this graphical summary, the maximum ages correspond with the largest SHAP values, and hence it is predicted that these patients are extremely likely to die as a result of COVID-19; for this reason, the sudden drop in the hospitalization and ICU age plots is likely a result of elderly patient fatalities occurring prior to admittance to hospital or ICU in the dataset. Largely, many severe cases of COVID-19 in Ontario during 2020 were associated with large numbers of residents in long-term care facilities passing away due to rapid spread among patients and personal care attendants (Liu et al., 2020). “Do-not-resuscitate” orders and the rapid decline of patients in these facilities are also potential reasons that create challenges in data in which fewer older patients have historically been admitted to the hospital or ICU. This provides an unanticipated result in the model predictions, since (as reported in the initial months of the pandemic), it is assumed that older patients frequently experience more serious outcomes from having COVID-19 (Niu et al., 2020).

The scatter plot distributions, in general, are intended to illustrate the significance of various comorbidities. While the SHAP plots indicate various features as less impactful than others (many having markers overlapping with the 0 SHAP line), the scatter plots can help better inform the analysis. Several features that have no influence on the predictions for those patients not identifying with the parameter might in reality be extremely significant comorbidities for those with the medical condition defined. As an example, the model is unlikely to modify any predictions for patients who do not have pneumonia, but for patients who *do* have pneumonia, this condition is likely to be considered by the model as a leading reason for severe illness or death, and hence acts as a comorbidity for those patients.

This further analysis suggests that pneumonia, weakened immune systems (being immunocompromised), underlying medical conditions, and cardiovascular disease are all predicted to be concerning comorbidities for those patients associated with these features (among others). In numbers, this may also apply to a smaller group of the population. Therefore, for the larger population, these attributes are of limited apparent concern but are fatally consequential for the smaller population.

Vaccination status is a highly relevant feature in all scenarios. Important to note is that the COVID-19 vaccines have been proven to decrease in effective protection over time (Howard, 2022)^1^. Therefore, while the model may predict SHAP values for the vaccinated/unvaccinated trait, a patient's time-since vaccination and the total number of doses are suspected to greatly impact their likelihood of becoming hospitalized, getting admitted to intensive care, or dying. It is anticipated that on the specific patient level, those who are recently vaccinated (14 days since inoculation) would be predicted to have a much more negative SHAP value than those who had been vaccinated for more than a period of 2 or 3 months (or longer).

From the data, it was calculated that of all recorded Delta cases, a higher percentage of the population was admitted to the hospital, ICU, or reported as a death when compared to the Omicron population. 4.650% of Delta cases were hospitalized, more than two times the hospitalizations noted for Omicron cases (at 2.096%). This calculation aligns with publicized reports that, despite being more transmissible, Omicron is less likely to cause severe illness in patients, unlike the previous dominant strain of the virus (Delta) (Vaughan, 2021).

### 5.1. Analysis of limitations and performance metrics

In epidemiology, the discussion related to “false negatives” and “false positives” is often associated with testing and modeling an infectious disease (Blair et al., 2009). In the context of this study, a false negative refers to a patient who was not predicted as hospitalized, admitted to ICU, or dead, but actually was one of these cases, in reality. Therefore, a false positive describes the situation wherein a patient is claimed to have been admitted to the hospital/ICU or died but was actually not associated with any of these scenarios.

From the perspective of decision-making and using modeling to advise on emerging medical risk factors that pose a potential insurgence to health care systems, it is ideal to minimize both forms of false prediction. However, a higher cost is associated with false negatives, as allocating resources for a smaller population that will inevitably exceed the resource threshold will cause a general overwhelming of the system. Over-accounting for potential hospital beds is less costly on the system than under-accounting, and therefore it is advised that models should have as low a count of false negatives as possible.

In artificial intelligence performance metrics, “recall” assesses the number of actual positive cases, and how many of these were properly predicted by the modeling (a “true positive,” predicted positive as well as actually positive) (Seliya et al., 2009). For this reason, a higher recall value indicates fewer false negatives and the most important metric in each of this study's models to be maximized. A maximized value for precision indicates fewer false positives, which is valuable in the authenticity of the model but not as vital for the application of the model in public health decision-making.

Table 3 provides the values of all performance metrics, including recall and precision. The highest value for the recall is 0.77, associated with the Delta variant predictions of hospitalization. The lowest value for the recall is 0.13, corresponding to the model for Omicron-related ICU predictions. Hence, while the results presented by the Omicron/ICU analysis align with the results in other scenarios, it should be noted that the inference confidence of this particular modeling scenario is lower than others. Similarly, from this recall calculation, it is appropriate to conclude that the model did not sufficiently capture all Omicron ICU entries, incorrectly labeling a significant number of the patients who were actually admitted as patients with non-intensive care.

Despite this discrepancy, the same model (Omicron/ICU) had the highest accuracy of all models, suggesting that the model addressed this scenario with the highest ratio of properly predicted (positive or negative) cases out of all possible cases. Overall the lowest accuracy of all models is the Delta/Hospitalization analysis at 0.85, albeit a relatively high value for accuracy.

The models generally have a higher number of false positives than false negatives, which is preferable. The lowest value for precision is 0.16 (although still higher than the lowest recall value) for Delta/ICU, and the maximum value is 0.23 for Omicron/Hospitalization. Hence, the difference between the “most precise” and “least precise” is marginal. This broadly suggests that all models are similar in incorrectly labeling non-serious patients as severe cases requiring advanced care.

The AUROC value in this context refers to the ability of the model to differentiate between Hospitalized/Non-Hospitalized, ICU/Non-ICU, and Death/Alive. The weakest model in this ability to discriminate between cases is the Omicron/ICU analysis with an AUROC of 0.57. This is in alignment with the aforementioned lowest recall being associated with Omicron/ICU predictions (it can be assumed that the model with the most false negatives might compete for the lowest ability to recognize cases appropriately). Conversely, the highest AUROC (0.81) is associated with the model that also has the highest value of recall; the Delta/Hospitalization predictions. The strength of this particular model suggests that it is highly effective at allowing for inferences and applications in public health settings, as resource allocation will generally be aligned with the potential demand for hospitalized care. As COVID-19 cases arise that are associated with highly implicated features defined by the modeling in Section 4 (cases of COVID-19 in persons with diabetes, pneumonia, and chronic illnesses), it is advisable to allocate more resources and hospital beds to account for the likely influx of patients requiring care.

## 6. Conclusion

An artificial intelligence-based approach to modeling and characterizing the trends in COVID-19 medical and personal risk factors comparing the differences between the two recent COVID-19 variants (Delta and Omicron) is a unique practice of pandemic research. XGBoost results proved that, consistently, age is the most impactful feature in predictions of hospitalization, ICU, and death outcomes from COVID-19. The older populations are routinely anticipated to develop more serious illnesses, with younger populations avoiding hospital care and death.

Patients who are unvaccinated or have diabetes, chronic kidney disease, complications of pneumonia, or cardiovascular disease, are shown to most frequently have higher significance in all scenarios, regardless of the variant. In most cases, the Delta and Omicron variants are consistent in being similar in their top 15 population features contributing to severe illness. This indicates that, although some discrepancies are still present (including that the Omicron variant is more transmissible), medical risk factors are similar across the board.

Finally, the Delta/Hospitalization model is the highest ranking application of AI for AUROC, F1, and Recall performance metric scores. Omicron/ICU and Omicron/Hospitalization have the highest accuracy and precision values, respectively. While many of the performance metric scores are large values (some reaching close to 0.99), the key metric, “recall,” was mostly above 0.60 with two exceptions. Suggested by this result is that the XGBoost model was successful at minimizing the number of false positives predicted, which would have less costly implications on a bigger scale (accounting for more of the people who would require medical care).

The results of this AI-based study present unique and significant findings on the intricate differences between hospital care and death factors among the Delta and Omicron variants. As the endemic phase of COVID-19 is embraced by an increasing number of jurisdictions, anticipating the need for future hospital beds will dictate the need for accurate and reliable modeling of the Ontario population. AI's role in decision-making in public health is an important and unparalleled aspect of designing a health care system that caters to the active and ever-evolving SARS-CoV-2 virus. As the virus continues to impact societies worldwide, comparisons and characterizations of variants of concern will steer the direction of health care, and advance disciplines in artificial intelligence.

## Data availability statement

The data analyzed in this study is subject to the following licenses/restrictions: This study was conducted using data sourced from ICES, which is funded by an annual grant from the Ontario Ministry of Health (MOH) and the Ministry of Long-Term Care (MLTC). Data access during the COVID-19 pandemic is overseen through the Ontario Health Data Platform (OHDP), a Province of Ontario initiative to address Ontario's ongoing response to the pandemic and its related impacts. Requests to access these datasets should be directed to https://www.ices.on.ca/DAS/Public-Sector/Access-to-ICES-Data-Process.

## Author contributions

EM, WH, BS, JY, and SG: conceptualization. WH: development and execution of methodology and software. MC: first draft of the manuscript and development of subsequent drafts. MC, EM, and WH: formal analysis. EM: supervision, project administration, and funding acquisition. All authors validation, writing, reviewing, and editing.

## Funding

The authors acknowledge the support of the Natural Sciences and Engineering Research Council of Canada (NSERC), (funding reference number 400677). Additionally, support from the NSERC Alliance COVID-19 fund (reference numbers 401636 and 401641) is also recognized. ICES, OHDP, and the staff at these organizations are recognized and appreciated for their data and guidance in the development of the research findings.

## Conflict of interest

JY was employed by Adastra Corporation. The remaining authors declare that the research was conducted in the absence of any commercial or financial relationships that could be construed as a potential conflict of interest.

## Publisher's note

All claims expressed in this article are solely those of the authors and do not necessarily represent those of their affiliated organizations, or those of the publisher, the editors and the reviewers. Any product that may be evaluated in this article, or claim that may be made by its manufacturer, is not guaranteed or endorsed by the publisher.

## Author disclaimer

This study was conducted using data sourced from ICES, which is funded by an annual grant from the Ontario Ministry of Health (MOH) and theMinistry of Long-TermCare (MLTC). Data access during the COVID-19 pandemic is overseen through the Ontario Health Data Platform (OHDP), a Province of Ontario initiative to address Ontario's ongoing response to the pandemic and its related impacts. The opinions, results, and conclusions reported in this paper are those of the authors and are independent of the data sources. No endorsement by the OHDP, ICES, its partners, or the Province of Ontario is intended or should be inferred. Parts of this material are based on data and/or information compiled and provided by Canadian Institute for Health Information (CIHI). However, the analyzes, conclusions, opinions, and statements expressed in the material are those of the author(s), and not necessarily those of CIHI. The use of the data in this project is authorized under section 45 of Ontario's Personal Health Information Protection Act (PHIPA) and does not require review by a Research Ethics Board.

## Figures and Tables

**Table:** 

**Variable/Abbreviation**	**Definition**
AUROC	Area under the receiver operating characteristic
ICES	The institute formerly known as the Institute for Clinical Evaluative Sciences, Ontario
ICU	Intensive Care Unit (level of hospitalization characterized as “critical care” for severe illness)
VIF	Variance inflation factor
VOC	COVID-19 variant of concern
ŷ_*k*_	The *k*^*th*^ “predicted” value for AI modeling
*y*_*k*_	The *k*^*th*^ “real” value for AI modeling

## Footnotes

^1^Hilal, W., Chislett, M. G., Snider, B., McBean, E. A., Yawney, J., and Gadsden, S. A. (2022). *A Survival Analysis Approach Evaluating the Likelihood of Testing Positive Post-Vaccination for COVID-19*. [Unpublished].

## References

[B1] Accorsi E. K. Britton A. Fleming-Dutra K. E. Smith Z. R. Shang N. Derado G.. (2022). Association between 3 doses of mRNA COVID-19 vaccine and symptomatic infection caused by the SARS-CoV-2 Omicron and Delta variants. JAMA 327, 639. 10.1001/jama.2022.0470 35060999 PMC8848203

[B2] Al-Tawfiq J. A. Hoang V.-T. Le Bui N. Chu D.-T. Memish Z. A. (2022). The emergence of the Omicron (B.1.1.529) SARS-CoV-2 variant: what is the impact on the continued pandemic? J. Epidemiol. Glob. Health 12, 143–146. 10.1007/s44197-022-00032-w 35089588 PMC8795715

[B3] Andrews N. Stowe J. Kirsebom F. Toffa S. Rickeard T. Gallagher E.. (2022). COVID-19 vaccine effectiveness against the Omicron (B.1.1.529) variant. N. Engl. J. Med. 386, 1532–1546. 10.1056/NEJMoa2119451 35249272 PMC8908811

[B4] Blair A. Saracci R. Vineis P. Cocco P. Forastiere F. Grandjean P.. (2009). Epidemiology, public health, and the rhetoric of false positives. Environ. Health Perspect. 117, 1809–1813. 10.1289/ehp.0901194 20049197 PMC2799452

[B5] Chen J. Wu L. Zhang J. Zhang L. Gong D. Zhao Y.. (2020). Deep learning-based model for detecting 2019 novel coronavirus pneumonia on high-resolution computed tomography: a prospective study. medRxiv. 10.1101/2020.02.25.20021568 PMC7645624 33154542

[B6] del Rio C. Omer S. B. Malani P. N. (2022). Winter of Omicron—the evolving COVID-19 pandemic. JAMA 327, 319. 10.1001/jama.2021.24315 34935863

[B7] Howard J. (2022). A Fourth COVID-19 Shot Might be Recommended This Fall, as Officials 'Continually' Look at Emerging Data. Atlanta, GA: CNN.

[B8] Huang C.-J. Chen Y.-H. Ma Y. Kuo P.-H. (2020). Multiple-input deep convolutional neural network model for COVID-19 forecasting in China. medRxiv. 10.1101/2020.03.23.20041608

[B9] Jin C. Chen W. Cao Y. Xu Z. Tan Z. Zhang X.. (2020). Development and evaluation of an artificial intelligence system for COVID-19 diagnosis. Nat. Commun. 11, 5088. 10.1038/s41467-020-18685-1 33037212 PMC7547659

[B10] Johnson A. G. Amin A. B. Ali A. R. Hoots B. Cadwell B. L. Arora S.. (2022). COVID-19 incidence and death rates among unvaccinated and fully vaccinated adults with and without booster doses during periods of delta and omicron variant emergence—25 U.S. Jurisdictions, April 4—December 25, 2021. Morb. Mortality Weekly Rep. 71, 132–138. 10.15585/mmwr.mm7104e2 35085223 PMC9351531

[B11] Khan A. Khan S. H. Saif M. Batool A. Sohail A. Khan M. W. (2022). A survey of deep learning techniques for the analysis of COVID-19 and their usability for detecting omicron. arXiv [Preprint]. arXiv: 2202.06372. 10.48550/ARXIV.2202.06372

[B12] Kumar P. Kalita H. Patairiya S. Sharma Y. D. Nanda C. Rani M.. (2020). Forecasting the dynamics of COVID-19 Pandemic in Top 15 countries in April 2020: ARIMA Model with Machine Learning Approach. Preprint, Health Inf. 10.1101/2020.03.30.20046227

[B13] Li L. Qin L. Xu Z. Yin Y. Wang X. Kong B.. (2020). Using Artificial intelligence to detect COVID-19 and community-acquired pneumonia based on pulmonary CT: evaluation of the diagnostic accuracy. Radiology 296, E65-E71. 10.1148/radiol.2020200905 32191588 PMC7233473

[B14] Li M. Zhang Z. Jiang S. Liu Q. Chen C. Zhang Y.. (2020). Predicting the epidemic trend of COVID-19 in China and across the world using the machine learning approach. Preprint, Epidemiol. 10.1101/2020.03.18.20038117

[B15] Liu M. Maxwell C. J. Armstrong P. Schwandt M. Moser A. McGregor M. J.. (2020). COVID-19 in long-term care homes in Ontario and British Columbia. Can. Med. Assoc. J. 192, E1540-E1546. 10.1503/cmaj.201860 32998943 PMC7721263

[B16] Madhi S. A. Kwatra G. Myers J. E. Jassat W. Dhar N. Mukendi C. K.. (2022). Population immunity and COVID-19 severity with Omicron Variant in South Africa. N. Engl. J. Med. 386, 1314–1326. 10.1056/NEJMoa2119658 35196424 PMC8908853

[B17] Nadeem M. F. Matti N. Parveen S. Rafiq S. (2022). Incessant threat of COVID-19 variants: highlighting need for a mix of fda-approved artificial intelligence tools and community pharmacy services. Res. Soc. Administ. Pharmacy 18, 3076–3078. 10.1016/j.sapharm.2021.07.018 34391673 PMC8294695

[B18] Niu S. Tian S. Lou J. Kang X. Zhang L. Lian H.. (2020). Clinical characteristics of older patients infected with COVID-19: a descriptive study. Arch. Gerontol. Geriatr. 89, 104058. 10.1016/j.archger.2020.104058 32339960 PMC7194515

[B19] Pourhomayoun M. Shakibi M. (2021). Predicting mortality risk in patients with COVID-19 using machine learning to help medical decision-making. Smart Health 20, 100178. 10.1016/j.smhl.2020.100178 33521226 PMC7832156

[B20] Rod J. E. Oviedo-Trespalacios O. Cortes-Ramirez J. (2020). A brief-review of the risk factors for COVID-19 severity. Revista de Saúde Pública 54, 60. 10.11606/s1518-8787.2020054002481 32491116 PMC7263798

[B21] Seliya N. Khoshgoftaar T. M. Van Hulse J. (2009). A study on the relationships of classifier performance metrics, in 2009 21st IEEE International Conference on Tools With Artificial Intelligence (Newark, NJ: IEEE), 59–66.

[B22] Snider B. McBean E. A. Yawney J. Gadsden S. A. Patel B. (2021a). Identification of variable importance for predictions of mortality from COVID-19 using AI models for Ontario, Canada. Front. Public Health 9, 675766. 10.3389/fpubh.2021.759014 34235131 PMC8255789

[B23] Snider B. Patel B. McBean E. (2021b). Insights into co-morbidity and other risk factors related to COVID-19 within Ontario, Canada. Front. Artif. Intell. 4, 684609. 10.3389/frai.2021.684609 34179769 PMC8222676

[B24] Snider B. Phillips P. MacLean A. McBean E. Gadsden S. A. Yawney J. (2020). Artificial intelligence to predict the risk of mortality from COVID-19: Insights from a Canadian Application. Preprint, Epidemiol. 10.1101/2020.09.29.20201632

[B25] Vaishya R. Javaid M. Khan I. H. Haleem A. (2020). Artificial Intelligence (AI) applications for COVID-19 pandemic. Diabetes Metab. Syndrome 14, 337–339. 10.1016/j.dsx.2020.04.012 32305024 PMC7195043

[B26] Vaughan A. (2021). Omicron emerges. New Scientist 252, 7. 10.1016/S0262-4079(21)02140-0 34876769 PMC8639363

[B27] Vilches T. N. Zhang K. Van Exan R. Langley J. M. Moghadas S. M. (2021). Projecting the impact of a two-dose COVID-19 vaccination campaign in Ontario, Canada. Vaccine 39, 2360–2365. 10.1016/j.vaccine.2021.03.058 33812742 PMC7980181

[B28] Wang S. Zha Y. Li W. Wu Q. Li X. Niu M.. (2020). A fully automatic deep learning system for COVID-19 diagnostic and prognostic analysis. Eur. Respirat. J. 56, 2000775. 10.1183/13993003.00775-2020 32444412 PMC7243395

[B29] World Health Organization (2022). Weekly epidemiological update on COVID-19-25 January 2022. Technical Report Edition 76.

[B30] Wu S. Yuan Q. Yan Z. Xu Q. (2021). Analyzing accident injury severity via an extreme gradient boosting (XGBoost) model. J. Adv. Transport. 2021, 1–11. 10.1155/2021/3771640

[B31] Yawney J. Gadsden S. A. (2020). A study of the COVID-19 impacts on the Canadian population. IEEE Access 8, 128240–128249. 10.1109/ACCESS.2020.3008608

